# Accuracy and Uncertainty of Gradient Based Leak Localization Procedure for Liquid Transmission Pipelines

**DOI:** 10.3390/s21155080

**Published:** 2021-07-27

**Authors:** Pawel Ostapkowicz, Andrzej Bratek

**Affiliations:** 1 Faculty of Mechanical Engineering, Bialystok University of Technology, Wiejska 45C, 15-351 Bialystok, Poland; 2 Łukasiewicz Research Network, Industrial Research Institute for Automation and Measurements PIAP, Al. Jerozolimskie 202, 02-486 Warsaw, Poland; andrzej.bratek@piap.lukasiewicz.gov.pl

**Keywords:** pipelines, leak localization, leak localization uncertainty, gradient method

## Abstract

This paper describes issues of leakage localization in liquid transmission pipelines. It focuses on the standard leak localization procedure, which is based on the calculation of pressure gradients using pressure measurements captured along a pipeline. The procedure was verified in terms of an accuracy and uncertainty assessment of the resultant coordinate of a leak spot. An important aim of the verification was to assess the effectiveness of the procedure in the case of localization of low intensity leakages with a level of 0.25–2.00% of the nominal flow rate. An uncertainty assessment was carried out according to the GUM convention. The assessment was based on the metrological characteristics of measuring devices and measurement data obtained from the laboratory model of the pipeline.

## 1. Introduction

Liquid transmission pipelines are notably exposed to leakage risks. The occurrence of a leakage usually leads to vast economic, environmental, and social effects.

Therefore, leak detection systems (LDS) are installed on transmission pipelines. LDS allows 24-h-a-day monitoring of the integrity of a pipeline, and the overall scope of operational tasks includes detection, localization, and estimation of a leak’s intensity.

In order to implement LDS, two categories of diagnostic methods are used [1,2,3]. The former ones, which detect leakages from outside of the pipe using special devices, i.e., microphones, hydrocarbon detectors, thermal cameras, are called external (direct or hardware based) methods. Others, which are based on measurements of flow parameters in a pipeline, such as the mass/volume flow rate, or the pressure and temperature, are called internal (indirect, analytical, or software based) methods.

Analytical methods appear to be of fundamental significance. Reviews can be found in [2,4]. In order to characterize any analytical method, it is worth noting that practical realization requires a combination of an appropriate measuring technique and an effective method of processing and analysis of the acquired signals. Among the analytical methods, we can note in particular quite simple solutions, such as mass or volume balance methods [5], negative wave pressure methods [4], or pressure point analysis methods [6]. Each of these methods deals with a different flow phenomenon, consisting in a specific flow rate and pressure changes in the pipeline, which are accompanied by the occurrence of leakage. In addition, more advanced analytical methods are utilized. In general, these are based on the use of a mathematical model of liquid flow dynamics in combination with computational techniques operating in real time. Examples of such methods are the compensated volume balance methods [4], which take into account line package variation due to changeability in the density of a pumped liquid medium, a cross section of the pipe, temperature, and pressure along the pipeline. In addition to this, the so-called automatic control approach methods [1,7,8] can be also mentioned, where the pumping process model is mainly described in a state space and a common solution for further analysis is an implementation of state observers.

In practice, any of the existing internal methods are not able to ensure all diagnostic tasks on their own. The use of these individual methods is also limited to specified pipeline operating conditions and leakage characteristics. Hence, in order to develop an effective and reliable LDS, it is necessary to choose and apply a few appropriate analytical methods [9].

Apart from the ability of LDS to detect leakage, another important issue is the precise localization of a leak point. In order to determine the coordinate where a leak has occurred, various localization procedures are used [4,10].

The most widely used are the negative pressure wave detection methods [4]. They use the phenomenon that accompanies the occurrence of a leakage, which involves the formation and propagation of negative pressure waves in a pipeline. These are initiated by a sudden drop in pressure at a leak point, which immediately propagates in both directions (downstream and upstream) through the pipeline as a wave. The most commonly used variant of these methods consists in tracking the moment of the wavefront [4,11]. The basis of its realization is the use of the signals acquired from a few or several dozen non-inertial sensors that are placed along the pipeline. Such variations can be implemented even using the diagnostic information only from two measurement points situated at the inlet and outlet of the pipeline. In the case of this variation, the leak point is calculated on the basis of moments, determined from the acquired signals, which correspond to the detection of pressure wavefront transition through a given measurement point. Generally, such techniques allow fast localization of a leak point; however, their use generates good results only in cases where pressure waves with clearly visible fronts arise due to an abrupt leak. These are not so obvious in the case of pressure waves with smoothed fronts, which arise as a result of a slowly increasing leak. Whereas, in the case of very slowly increasing leaks, such procedures can be practically useless.

The occurrence of a leak, in addition to the above-mentioned phenomenon of pressure wave propagation, is also accompanied by specific changes (drops) in pressure values along the pipeline, as well as changes in flow streams in the upstream and downstream sections of the leak. The pressure drop is greatest at the leak point and decreases as distance from the point increases. Pressure point analysis methods [6] are an example of using the phenomenon of pressure drops in a pipeline to locate a leak. The implementation of these methods is based on the use of signals obtained from sensors located at specific points in the pipeline, the assessment of which is aimed at identifying the highest values of pressure drops. In practice, this consists of comparing the current mean pressure values for certain measuring points with their previously estimated values, and the differences, obtained in this way, are compared with the defined threshold values. In the simplest variants of such rough methods, flow models are not used, and the nominal pressure values estimated for individual measurement points are statistically calculated on the basis of the obtained data samples. In the case of such variations, the leakage location is also not precisely identified. This kind of assessment is only a rough one. By identifying measuring points with the highest pressure drops and their proximities to each other, in the end, the pipeline section where a leak occurs is identified. Despite such limitations, these simplified solutions can be very useful, especially when they are properly combined with other techniques.

However, thanks to the pressure drop in the pipeline, it is possible to obtain the exact location of a leak based on the calculation of pressure gradients [12,13,14,15]. Gradient-based procedures are used both in simplified [16] as well as very advanced solutions [1,8] of analytical methods. Such procedures are used for diagnosing leaks when they occur in both steady and transient states, i.e., related to an operating point change, a valve’s aperture and closure, and pump start-up or stoppage. Gradient-based leak localization procedures are not fast as localization procedures based on negative pressure wave detection [17]; however, compared to those procedures, they are more reliable, i.e., offer a smaller risk of missing a leak.

When considering any of the leak localization procedures, the basic measure of its effectiveness is the accuracy of the obtained leak location coordinate. Pipeline operators expect a high level of accuracy, i.e., as little location error as possible, even in the case of very small leaks. Their expectations also relate to leakages of less than 1% of the nominal flow rate. In practice, determining the exact location of such small leaks is quite a challenge.

Apart from information about the error value, an important issue is the uncertainty of the leak localization’s results, which are expressed numerically in units of length.

With any method of locating a leak, its effectiveness depends on many factors. In the overall approach of the implementation of a given method, the following should be noted: configuration of measurement devices (their number, location), their metrological characteristics (precision), signal sampling, measurement conditions (noise and distortion level), as well as the calculation algorithm that covers, not only the main computational procedure of the results, but also certain techniques implemented for data processing and analysis, as well as the selection of alarm thresholds if they are needed. Other factors involve a pipeline and pumping process, among which the following are significant: topography (e.g., diameter and length), type of pumped liquid (density, velocity of a pressure wave), and flow conditions during the occurrence of a leak (steady or transient state, values of flow and pressure). Moreover, the leak parameters are also essential, such as its location, size (intensity) or the nature of occurrence (rapid leaks or slowly increasing leakages).

Awareness of these aspects, as well as their impacts on the effectiveness of leak localization, enables proper selection of the most optimal variant of a given method. When making such a selection in practice, the costs associated with the installation and operation of the LDS, within the scope intended for use of this method, are usually significant limitations. Essentially, while estimating the effectiveness of leak localization through numerical values of the above-mentioned qualitative indicators, they may be useful in the case of undertaking steps to further improve a given method or even a specific variant. Based on these, you can also make comparisons between different leak localization methods.

For the two qualitative indicators mentioned, however, the information available in the literature concerns only accuracy. According to the authors, disregarding the uncertainty of calculated leakage point results is not fully justified. Uncertainty should be treated as valuable additional information. It can be very helpful for pipeline operators, taking action in response to a leakage alarm signaled by an LDS, as an indication of the potential coordinates of a leak’s location. In particular, this applies to decisions, such as closing certain valves in order to cut off the damaged section of the pipeline and sending repair teams to the site and indicating the scope of the search for the location of the damaged pipeline.

Hence, the motivation of the author of this work is to estimate both the accuracy and uncertainty of the standard gradient leak localization procedure. The uncertainty of the leak localization result is considered here in the context of estimating systematic and random measurement errors and their impacts, through a mathematical relationship, which defines the main leak location calculation procedure. Other parameters included in the calculations are also taken into account, as is the impact on the final results of their determination uncertainty. As a result, this leads to a numerical determination of the tolerance of leak location results, i.e., the boundaries of the range within which it can be found.

We propose to assess the uncertainty in accordance with the GUM convention, using methods commonly known in metrology, described in the guide [18] and in the ISO 5168 standard [19], which concerns the implementation of GUM in the domain of flow metrology.

We also assume that acquired measuring data are free from the field instrumentation defects or discontinuities of data transmission. Such elements are important in case of another quality indicator: robustness. While assessing the resistance of any leak localization procedure, other issues should also be taken into account, such as changes of input variables values (measured signals) or pipeline operating conditions and their impacts on the leak location results. However, these are not in the scope of this work.

This work’s contribution consists in the presentation and discussion of issues related to uncertainty estimation using the example of the implementation of a standard gradient-based leak localization procedure, which uses measurement data acquired from the laboratory model of the pipeline. In order to assess the uncertainty, characteristics of measuring devices applied on the pipeline are taken into account. The scope of the operational area of this procedure concerns single low intensity leaks of about 0.25–2.00% of the nominal flow rate. Three different leak spots situated at the inlet, middle, and outlet sections of a pipeline are considered. Moreover, such an assessment is realized in reference to different variations of the procedure, including a variety of pressure sensor pair configurations used for the calculation of pressure gradients and a number of data samples used for estimation of the nominal values of particular measured pressures. The obtained results were also supplemented with an exemplary uncertainty budget, which shows the impact of individual input parameters on the uncertainty of the results. Such information may be useful to further improve the effectiveness of the procedure under study. We also present an analysis of the obtained results.

This paper is organized as follows: the second section presents a description of the standard gradient-based leak localization procedure, including requirements related to its application. Definition of the accuracy and the way in which the uncertainty of a leak point is calculated by using the discussed procedure is evaluated, taking into account type A and B measurement uncertainties, are also in this section. The third section presents the laboratory water pipeline and experiments’ program overview. In this section, aspects of the in-practice implementation of the procedure in question are discussed. The next section presents results achieved in the field in terms of the accuracy and uncertainty of the examined procedure, together with their discussion. Finally, conclusions are presented.

## 2. Materials and Methods

### 2.1. The Standard Gradient Based Leak Localization Procedure

The accuracy of leak localization for any internal method is characterized by the error, i.e., the difference between the estimated and real place of the leak.

The assessment of uncertainty of any internal method should consider all main components, which correspond to the method’s definition according to the diagnostic theory.

Therefore, components, such as measuring instruments of process variable signals, process diagnostic models or algorithms and calculation formulas used to obtain a diagnosis, should be involved.

#### 2.1.1. Pipeline Measuring Equipment and Measurement Data Quality

Liquid transmission pipelines are equipped with sensors to monitor flow process. When using such sensors for the purpose of leak detection and localization realized with analytical methods, the following parameters should be determined:

− pressure at the inlet $p_{in}$, at the end $p_{out}$ and at several points along the pipeline;
− volumetric (or mass) flow rate, at least at the inlet $q_{in}$ and/or at the outlet $q_{out}$ of the pipeline;
− additionally, others parameters, i.e., temperature at the inlet and outlet and ground temperature, as well as the state of control devices (pump, valves) to monitor changes of operational conditions in the pipeline.

These measured signals are never perfect, but include noise and systematic measurement errors, as a result of the complex and extensive structure of the measuring systems applied to such pipelines. In addition, measuring data can be falsified by disturbances that occur in the flow [20].

#### 2.1.2. Computational Formula Used for Leak Location

Standard gradient-based leak localization procedures [12,13,14] take advantage of accompanying leakage pressure changes (drops) in the pipeline. Such procedure may be useful for leaks that occur at one or even several locations along the pipeline. In this work we were interested in the localization of only a single leak.

**Figure 1:**
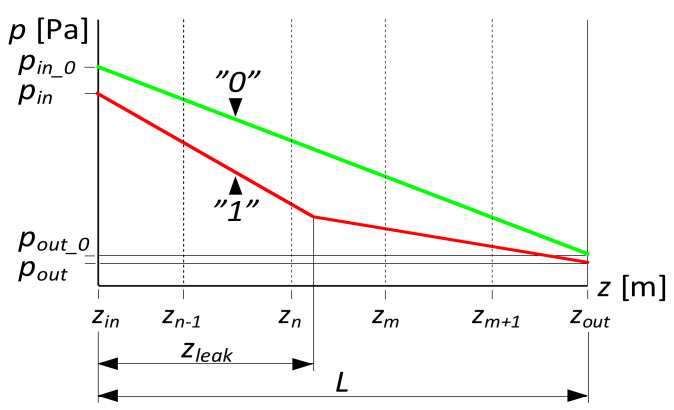
Lines of pressure distribution during no-leak conditions and after leak occurrence.

The procedure uses the phenomenon of the change in the pressure distribution along the pipeline, after leak occurrence (Figure 1). It consists in the calculation of the abscissa of the intersection point of two straight lines, which is shown in Figure 1 (curve “1”), using the following relationship:

(1) $$z_{leak}=\frac{p_{out}-p_{in}-G_{out}\cdot L}{G_{in}-G_{out}}$$

where L—length of the examined pipeline’ section, $G_{in}$, $G_{out}$—pressure gradients after the leak occurrence in the subsection between the beginning of the examined section and the leak point and in the subsection between the leak point and the end of the pipeline section, and $p_{in}$, $p_{out}$—pressure after the leak occurrence for the initial and final cross-section of the examined pipeline section.

Both lines, which are related to the pressure distribution upstream and downstream of a leak, can be described as:

(2) $$p(z<z_{leak})=p_{in}+G_{in}\cdot z$$

(3) $$p(z>z_{leak})=p_{out}-G_{out}\cdot L+G_{out}\cdot z$$

In order to determine gradients $G_{in}$ and $G_{out}$, it is necessary to use at least four pressure sensors, located in the examined pipeline’s section, two in front of and two behind the leak point.

For both pairs of pressure sensors, it is preferable to use those that are located as far from each other as possible.

Both pressure gradients, $G_{in}$ and $G_{out}$, can be calculated using the following relationships:

(4) $$G_{in}=\frac{p_{n}-p_{in}}{z_{in,n}}$$

where $p_{in}$—pressure at the inlet of the examined pipe’s section, $p_{n}$—pressure at a point located in front of the leak point, $z_{in,n}$—distance between both points.

(5) $$G_{out}=\frac{p_{out}-p_{m}}{z_{m,out}}$$

where $p_{out}$—pressure at the outlet of the examined pipe’s section, $p_{m}$—pressure at a point located behind the leak point, $z_{m,out}$—distance between both points.

#### 2.1.3. Requirements Related to the Procedure Application

Generally, gradient-based leak localization procedures do not require pressure signals to be sampled at a very high rate, which is necessary in the case of localization procedures based on the negative pressure wave method. In real pipelines, sampling periods range from a few seconds to several minutes.

However, gradient-based leak localization procedures need to use data from high accuracy pressure sensors. It is necessary to get an accurate estimation of the nominal values of the measured pressures that determines the precision of the leak localization.

In practice, the nominal values of the measured pressures cannot be estimated on the basis of a single data sample. This is due to the previously mentioned fact that measured pressure signals in liquid transmission pipelines usually contain noise disturbances and interference caused by flow and measurement effects. Therefore, the estimation of the nominal values of the individually measured pressures requires the use of identical measurement time windows covering a certain number of data samples.

Averaging each of these sets of data samples is applied here, i.e., their average values are calculated [21,22]. As is known, the accuracy of averaging increases with the amount of available data [23,24]. On the other hand, it means increasing the total detection time (response time).

### 2.2. The Uncertainty of the Leak Localization Procedure

The uncertainty of the leak location procedure (1) will be determined by a combined uncertainty, what corresponds to the standards introduced in [18]. We assume that the input quantities, which are measured pressures $p_{in}$, $p_{n}$, $p_{m}$, $p_{out}$, are uncorrelated.

Before the uncertainty of discussed procedure can be determined, it is possible to estimate the uncertainties of both gradient calculations, $G_{in}$ and $G_{out}$, in the beginning, defined by dependencies (4) and (5).

The $G_{in}$ gradient uncertainty can be calculated using the following formula:

(6) $$u\left(G_{in}\right)=\sqrt{{\left[u\left(p_{in}\right)c\left(p_{in}\right)\right]}^{2}+{\left[u\left(p_{n}\right)c\left(p_{n}\right)\right]}^{2}+{\left[u\left(z_{in,n}\right)c\left(z_{in,n}\right)\right]}^{2}}$$

where the individual sensitivity coefficients c(…) can be calculated as partial derivatives (4), that is:

(7) $$\begin{array}{l} c(p_{in})=\frac{\partial G_{in}}{\partial p_{in}}=-\frac{1}{z_{in,n}}\\ c(p_{n})=\frac{\partial G_{in}}{\partial p_{n}}=\frac{1}{z_{in,n}}\\ c(z_{in,n})=\frac{\partial G_{in}}{\partial z_{in,n}}=-\frac{p_{n}-p_{in}}{z_{in,n}^{2}} \end{array}$$

Identically, the $G_{out}$ gradient uncertainty can be calculated:

(8) $$u\left(G_{out}\right)=\sqrt{{\left[u\left(p_{out}\right)c\left(p_{out}\right)\right]}^{2}+{\left[u\left(p_{m}\right)c\left(p_{m}\right)\right]}^{2}+{\left[u\left(z_{m,out}\right)c\left(z_{m,out}\right)\right]}^{2}}$$

where the sensitivity coefficients c(…) as partial derivatives (5) are as follows:

(9) $$\begin{array}{l} c(p_{out})=\frac{\partial G_{out}}{\partial p_{out}}=-\frac{1}{z_{m,out}}\\ c(p_{m})=\frac{\partial G_{out}}{\partial p_{m}}=\frac{1}{z_{m,out}}\\ c(z_{m,out})=\frac{\partial G_{out}}{\partial z_{m,out}}=-\frac{p_{out}-p_{m}}{z_{m,out}^{2}} \end{array}$$

Now, by differentiating (1), individual sensitivity coefficients can be determined:

(10) $$\begin{array}{l} c(L)=\frac{\partial z_{leak}}{\partial L}=\frac{-G_{out}}{G_{in}-G_{out}}\\ c(G_{in})=\frac{\partial z_{leak}}{\partial G_{in}}=\frac{-(p_{out}-p_{in}-G_{out}\cdot L)}{{\left(G_{in}-G_{out}\right)}^{2}}\\ c(G_{out})=\frac{\partial z_{leak}}{\partial G_{out}}=\frac{p_{out}-p_{in}-G_{in}\cdot L}{{\left(G_{in}-G_{out}\right)}^{2}}\\ c(p_{in})=\frac{\partial z_{leak}}{\partial p_{in}}=\frac{-1}{G_{in}-G_{out}}\\ c(p_{nut})=\frac{\partial z_{leak}}{\partial p_{out}}=\frac{1}{G_{in}-G_{out}} \end{array}$$

and the formula defining the uncertainty of the standard leak location procedure is given as follows:

(11) $$u\left(z_{leak}\right)=\sqrt{{\left[u\left(L\right)c\left(L\right)\right]}^{2}+{\left[u\left(G_{in}\right)c\left(G_{in}\right)\right]}^{2}+{\left[u\left(G_{out}\right)c\left(G_{out}\right)\right]}^{2}+{\left[u\left(p_{in}\right)c\left(p_{in}\right)\right]}^{2}+{\left[u\left(p_{out}\right)c\left(p_{out}\right)\right]}^{2}}$$

The considered method, as well as the dependence (11) itself, correspond to the uncertainty propagation law described as (12). In order to estimate u(y), the combined uncertainty of a function of several variables designated conventionally as $x_{i}$, the so-called sensitivity coefficients $c_{i}$, calculated with the operator’s calculus, were used. However, we should bear in mind that the individual sensitivity coefficients ($c_{i}\equiv dy/dx_{i}$) correspond to the assumption of a linear relationship between the uncertainty of individual input variables, $u(x_{{}_{i}})$, and their corresponding uncertainty components $u_{i}(y)$. Finally, it may result in a certain degree of approximation of the result of u(y).

(12) $$u^{2}(y)=\sum_{i=1}^{N}{\left[c_{i}u(x_{i})\right]}^{2}\equiv \sum_{i=1}^{N}u_{i}{}^{2}(y)$$

Another way to estimate the uncertainty of a result, which may also be used as a solution’s validation, is the so-called incremental method represented by relationship (13). It consists in determining the resulting function’s increments from the changes in individual input variables, corresponding to uncertainty $u(x_{{}_{i}})$, where such a component is a measure of uncertainty $u_{i}(y)$. This method makes it possible to check whether the second-order nonlinearity should also be taken into account in terms of the relationship between the components of uncertainty result $u_{i}(y)$ and the influence of the uncertainty of individual input variables, $u(x_{{}_{i}})$.

(13) $$u_{i}(y)=\left|y(x_{i}+u(x_{i}))-y(x_{i})\right|$$

The uncertainty estimation results obtained using both techniques usually do not differ significantly.

It should be noticed here that, in the calculations of the both pressure gradients $G_{in}$ and $G_{out}$, and finally the coordinate of the leak point, $z_{leak}$, we used the estimated values of the individual pressures $p_{in}$, $p_{n}$, $p_{m}$, $p_{out}$. Each is determined on the basis of repeated pressure measurements, i.e., using a given set of N data samples, for which the average value is calculated.

Therefore, the standard uncertainties corresponding to these pressure variables should be estimated, taking into account the standard uncertainty of type A, as well as the standard uncertainty of type B [25,26]. Such individual uncertainties can be determined according to the following relationship:

(14) $$u(p)=\sqrt{{\left[u_{A}(p)\right]}^{2}+{\left[u_{B}(p)\right]}^{2}}$$

#### 2.2.1. Measurement Uncertainty of Type A

The type A uncertainty relates to accidental measurement errors. Such standard uncertainties of the individual pressure variables $p_{in}$, $p_{n}$, $p_{m}$, $p_{out}$ can be estimated on the basis of statistical analysis of their corresponding set containing N measuring data samples. For each of these variables, the estimator of the standard deviation of the mean $\bar{p}$, described by Formula (15), can be used as the type A uncertainty measure.

(15) $$u_{A}(p)\equiv s(\bar{p})=\sqrt{\frac{\sum_{r=1}^{N}{\left(p_{r}-\bar{p}\right)}^{2}}{N(N-1)}}$$

#### 2.2.2. Measurement Uncertainty of Type B

The type B uncertainty relates to systematic measurement errors. An useful measure of the systematic error is the limiting uncertainty Δp, i.e., the range (p−Δp,p+Δp), in which real measurement values are contained. The limiting uncertainty is recalculated to the standard uncertainty. Such standard type B uncertainties corresponding to the individual measured pressures $p_{in}$, $p_{n}$, $p_{m}$, $p_{out}$ can be expressed by relationship (16), where a specific distribution of systematic errors is also assumed and that is represented by the coefficient $\sqrt{a}$.

(16) $$u_{B}(p)=\frac{\Delta p}{\sqrt{a}}$$

The most used solution is the estimation of the band of Δp on the basis of the maximum absolute measurement error. The limit of such an error can be calculated using information given in the manufacture’s manuals of the measuring devices.

When considering the measuring system of a given pipeline, a hardware structure of individual channels employed for each of the measured pressures is crucial [27]. It is recommended here to consider, not only pressure transmitters, but also other components of these measuring channels, including A/D converters, communication modules for wire or no-wire data transmission, cables, etc.

### 2.3. Experimental Verification of the Standard Gradient Based Leak Localization Procedure

The considered leak localization procedure has been put into experimental tests. In the research, the laboratory model of a pipeline for pumping water was used.

#### 2.3.1. The laboratory Pipeline

**Figure 2:**
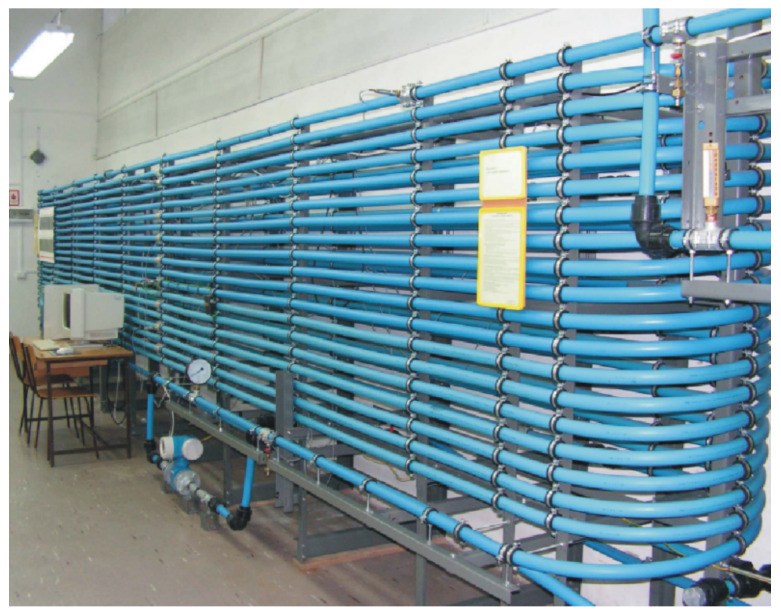
Laboratory model of the pipeline used during experiments.

The laboratory test stand (Figure 2) is located in the Faculty of Mechanical Engineering of the Bialystok University of Technology in Poland. Its main part is the pipeline. The total length of the pipeline is close to 400 m, including the main pipe section which is 380 m long and is made of polyethylene tubes (HDPE) with a 34 mm internal diameter and 40 mm external diameter. Moreover, the laboratory test stand consists of a variable flow pump, two semi-open tanks (at the inlet and outlet) each with a 300 dm^3^ capacity.

The pipeline is equipped with measuring devices, i.e., two electromagnetic volume flow meters (at the inlet and outlet) and several pressure sensors along the pipe, as well as thermometers. Sensors are connected to a PC using 16-bit A/D converter.

In order to simulate leakages, solenoid valves equipped with interchangeable diameter orifices are used. They are installed at selected points along the whole length of the main pipeline section.

#### 2.3.2. Positions and Metrological Characteristics of Pressure Sensors

**Figure 3:**
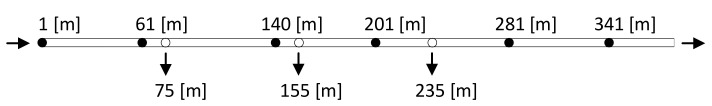
Pressure transmitters and leak point positions.

Figure 3 shows a diagram of the pressure transmitter locations and the single leak positions configured during the experiments.

Identical maximum absolute errors were assumed for all measured pressure signals, which were acquired from the individual transmitters located on the pipeline. The upper and lower limits of these errors were equal to Δp=1.20 kPa. Such a value was determined considering two main components of measuring systems, i.e., the pressure transmitters and the A/D converter. In order to make this calculation, information available in the manufacture’s manuals of these devices was used.

It was also assumed that the pressure measurement error distribution was concentrated around the middle of the interval, where a triangular error distribution was considered. Hence, it is acceptable to express type B standard uncertainty of measured pressure using $u_{B}(p)=\Delta p/\sqrt{6}$. For comparison, the uncertainty of type B for a uniform error distribution is expressed by $u_{B}(p)=\Delta p/\sqrt{3}$. Assuming such a distribution, it usually leads to overstating of standard uncertainty assessment.

Moreover, for all distances considered in Equations (1), (4) and (5), their standard uncertainty was assumed to be 0.025 m.

#### 2.3.3. Conditions of Experiments

During the experiments, the pipeline was operated in a steady state prior to each simulated leak. Individual leaks were simulated at three previously defined points of 75, 155 and 235 m coordinates. All leaks were initiated by a sudden opening of solenoid valves using a step change in the control signal. The leak sizes ranged from around 0.25% to around 2.00% of the nominal flow rate value. The pressure was sampled with a frequency $f_{P}=100$ Hz. The temperature of the pumped water ranged from 15 °C to 25 °C.

#### 2.3.4. Practical Implementation of the Procedure, including the Choice of Pressure Sensor Pair Configuration and the Calculation of the Average Pressure Values

In practice, the leak localization procedure (1) is triggered once an alarm is raised by the leak detection algorithm. However, in the research we only proposed to focus on localization. We assumed that, for all the experiments, the discussed leak localization procedure would be activated after 5 s, counted from the moment of leak occurrence. Such a time value corresponds to the results of the experimental studies presented in [15,16], where the algorithms designed to detect a leak were tested for similar scenarios of leak occurrence.

Computing the leak’s position requires data obtained for individual measurement points. For considered the leak localization procedure, these data correspond to the state with a leak. In addition, it is assumed that such a state will correspond to the pipeline operating under steady state conditions. This assumption facilitates the uncertainty analysis, because the mean values of the measured pressure will be calculated.

For all experiments, we performed calculations using the individually measured pressure signals after the reduction in the sampling frequency to a level equal to $f_{P}=10$ Hz.

In this research, different configurations of two pairs of pressure transmitters, i.e., the initial pair and final pair, were used to calculate pressure gradients in the leak localization Formula (1). Three variants of such pairs were used (see Table 1):

− extremely located sensors (marked as “A”),
− sensors closest to the leak point (marked as “B”),
− extremely located sensors and those closest to the leak point (marked as “C”).

A correct determination of the pressure measurement points, which are considered in the gradient calculations, is crucial.

Given that the pipeline leaks are between the extreme sensor pairs, which refers to the configuration marked "A", this decision does not need to be made. However, it is required in the case of the “B” and “C” configurations of sensors pairs (see Table 1).

In order to identify sensors that would correspond to the “B” and “C” configurations, the solutions proposed and tested by the authors may be quite helpful.

Ostapkowicz [15] proposed a solution based on the map of gradient increments, $\Delta G_{p,p-1}$, calculated between each pair of successive pressure measurement points, located along the pipeline, defined for i=0,…,j, where p=1,…,j. The increments for each gradient, which take into account the gradient values before and after the leak occurrence, are determined as:

(17) $$\Delta G_{p,p-1}=G_{p,p-1}-G_{p,p-1(0)}$$

Within the instant of leak detection by the detection algorithm, the map calculations are synchronized and triggered. The analysis of such a map, starting from the beginning of the pipeline, consists in observing the value change of adjacent gradient increments, from negative to positive.

Bratek proposed a more complex set of calculation procedures, whose detailed description might be found in [17]. Assuming that pressure sensors, successively installed along the pipeline, were similarly defines as i=0,…,j. At the beginning, only the potential leak area is indicated, linked to the leak detection event by the procedure $B_{0}$, which use the flow rate measurements $q_{in}$ and $q_{out}$, as follows:

(18) $$B_{0}(t)=q_{in}(t)-q_{out}(t)$$

The result of rough leak detection is a determination of the pressure sensor number i=k for which the indicator function, IF, calculated for additional procedure, $AR_{k}$, achieved its minimal value $AR_{k}\left\{IF\right\}$ procedure is based on the analysis of the deviations (residuum), according to formula (19). Such residuum is calculated using the pressure measured in the determined point, i=k (0<k<j), and the value modeled at this point. The model is considered as a pipeline’s pressure distribution based on the measurements: $p_{in}$ and $p_{out}$.

(19) $$AR_{k}(t)=p_{k}(t)-{\hat{p}}_{k}(t)$$

where ${\hat{p}}_{k}$—estimated pressure value at measurement point k.

The result of this procedure is the determination of pressure sensors i=k−1 and i=k+1 situated as close as possible to sensor i=k on both sides.

This procedure was successfully used for leakage diagnosis in a transient state. Experimental tests scenarios involved the simulation of leaks during changes in the pipeline’s operating conditions, consisting in increasing the pump’s rotation velocity [17].

Both solutions take a significant step towards sensors selection for pairs of “B” and “C” configurations; however, we need to take into account that additional correction might still be required in the case of determining adjacent gradient increments or indicating sensors located on both sides of a fixed sensor. The aim of this correction is to directly indicate sensors located closest to the leak from both sides.

In further considerations, we assumed that the leak location for the “B” and “C” configurations was based on the known conditions of the performed experiments.

Moreover, in the research, three variants of sets were used in order to calculate the nominal values of the individual measured pressures; $p_{in}$, $p_{n}$, $p_{m}$, $p_{out}$ were considered. These sets were different in a number, N, of data samples, which amount to: N=10, N=100, and N=500.

## 3. Results and Discussion

### 3.1. Accuracy Assessment of the Standard Gradient Based Leak Localization Procedure

Table 2 presents simulated leak location errors obtained for the discussed procedure, based on dependency (1). The results concern the use of “A”, “B”, and “C” sensor pair configurations, as well as sets including N=10, N=100 and N=500 data samples, which were used in order to estimate the nominal values of the individual measured pressures.

In addition, Table 3 presents the averaged absolute values of the location errors for each leak location (marked as ${\left|error\right|}_{75}$, ${\left|error\right|}_{155}$ and ${\left|error\right|}_{235}$) and the averaged value for all of the locations together (marked as ${\left|error\right|}_{all}$).

We determined that the presented results of the uncertainty estimation $u(z_{leak})$, obtained on the basis of dependence (11), in the range of the set approximation level of 0.1 m, show practically no differences in the case of using dependence (13).

It should be explained here that the leak location errors concerning the individual experiments show average values. These were obtained on the basis of the errors corresponding to the leak point calculations obtained from the pressure data from three distinct time windows. The used time windows, which contained an identical number of data samples, were shifted in turn by half the length of a single window. The start of the first window was positioned with the previously mentioned interval of 5 s, counting from the moment of simulating the leak.

According to the statistics rules, such orders of time windows can provide a less reliable result of leak localization than in the case of using pressure data from time windows, which do not overlap. Assuming the possibility of obtaining an accurate result of the leak location, such a window arrangement, compared to the former arrangement, shortens the diagnosis time. The use of such a window system can also be justified by the greater degree of protection of measurement data, in the event of such situations such as slow changes in flow parameters in a pipeline or a measurement drift [28]. It is known that their negative impact increases with the extension of the measurement recording period. In practice, they will distort the results of statistical calculations, which relate to measurement data captured in a given time window, including the calculated average value of such a set.

It should also be kept in mind that the discussed procedure imposes certain limitations to the credibility of leak localization results. Theoretically, the calculated leakage location value, according to Equation (1), should only fall within a certain range. The boundaries of such a range are defined by the coordinates of the location of the pressure sensors, which are taken into account in the calculations of both pressure gradients.

For individual experiments, when the calculated localization error, corresponding to the measurement data for any of the time windows, did not meet such conditions, the cases were marked in bold.

Moreover, when calculating the leakage location according to dependency (1), the coordinates of the main section of the pipeline were set as the limit values of the results. When the calculated leakage spot exceeded the length of the pipe section, the inlet or outlet coordinate was selected accordingly as the leak location. For individual experiments, if such cases were found for any of the time windows, they were marked in bold.

While analyzing the presented results, it should be considered that they include absolute errors. From a general perspective, one can observe that the values obtained for the individual experiments were quite varied, ranging from the tenth part of a meter up to a few hundred meters.

The absolute error values clearly determined the accuracy of the leak location. However, in order to be able to correctly interpret such results, they should be considered in relation to the scale of the pipeline being diagnosed. This applies, in particular, to the case when the object to be diagnosed is a model pipeline, of which the length is approximately four hundred meters.

Taking the above into account, the following assessments characterizing the accuracy of the leak location can be assigned for the error levels given below:

− up to several meters as “good”;
− about a dozen or so meters as “acceptable”;
− around several dozen meters as “unsatisfactory”;
− hundreds of meters as “negative”, which should be treated as impossible for finding a leak position.

In addition to using the above key points to evaluate the localization results obtained for the compared variants of the considered procedure, they should also be assessed by taking into account two parameters characterizing the simulated leakage, i.e., location and size (intensity).

Analyzing the leak location errors obtained for the discussed procedure, which correspond to the examined variants of its implementation, one can observe that:

- The individual pressure sensor pair configurations, “A”, “B”, and “C”, result in clear differences between error values.
  In the case of the examined pipeline, it allowed us to choose the optimal pressure sensor pair configuration. Considering the results obtained for the experiments with exactly the same simulated leak location and within the overall approach, the most precise leak location detection was ensured by configuration “C”. In the case of this configuration, a decrease in localization error value as the leak size increased could also be noticed. This only applies to leakages that were simulated in the initial section or partially in the middle of the pipeline. Such dependencies were not observed for the other two configurations of sensor pairs. The ”C” configuration was based on the calculation of both gradients, $G_{in}$ and $G_{out}$, by considering the greatest distances between the individual pressure sensors. In addition, the internal sensors of both pairs were located closest to the leak. Both of these factors resulted in a more accurate determination of the slope of the lines, as well as their intersection point, which determines the coordinate of the leak location. This is crucial in the case of low intensity leakages, where the values of both pressure gradients, i.e., the slope of both lines, do not vary much.
  The worst results were obtained for the remaining configurations: “A” and “B”. The “A” configuration turned out to be less effective in the case of leaks that occurred at the initial and final pipeline sections. This is caused by the fact that both gradients, $G_{in}$ and $G_{out}$, were calculated on the basis of the extreme pairs of pressure sensors mounted at the inlet and outlet of the pipeline. In addition, it also was caused by the smaller distances between the sensors in both pairs, as well as by the large distances between the leakage point and the internal sensor from the sensor pair located on the opposite side of the pipeline. In the case of configuration “B”, the worst results were obtained for leakages in the middle section of the pipeline. This configuration was based on gradients calculated for the sensor pairs closest to the leak and, in some experiments, the leak location errors even exceeded the extreme coordinates defining the length of the tested section of the pipeline.
- The following number, N=10, N=100 and N=500, of data samples used for the estimation of nominal values of the individual measured pressures did not produce clearly visible effects between the accuracy of the localization of a leak point, but such significant differences can be noticed for the dispersion of a leak location error.
  Here, we should focus on determining whether and what benefits, in terms of accuracy of the leakage localization, result from increasing the number of N data samples, whose mean value estimates the measured pressure. In addition, it is also important to identify mutual differences between the use of sets with the considered numbers of N data samples.
  Based on the results obtained for individual experiments, which correspond to the use of identical variants of pressure sensor pairs, it cannot be noticed that, with the increase in the number of N data samples for each of the experiments, the leakage localization error was reduced. This applies to practically all the considered sensor pair configurations. However, it can be observed that, as the number of N data samples increases, the number of the most undesirable results decreases, which is marked with bold characters.
  Moreover, another difference can be observed for the averaged absolute values of the location errors obtained for each leak location and is the averaged value for all of the locations together. It is worth mentioning that these results represent the average error spread. When we consider such averaged absolute values of location errors obtained for each leak location, for each of the considered sensor pair configurations, an improvement in the spread of location errors could only be seen for some of the three leak simulation sites considered. More visible differences can be noticed while analyzing the averaged absolute values of location errors in reference to each leak location together. Along with an increase of the number N data samples, only the results corresponding to the pressure sensor pair configurations “B” are not characterized by an improvement in the accuracy of the leak location. In the case of configuration “A”, we can observe a decrease in the dispersion of the leak location error. However, such differences between the results corresponding to the considered sets amount to one meter.
  On the other hand, the greatest scope of improvement of such results, i.e., around a few meters, was obtained in the case of the “C” sensor pair configuration. With regard to this configuration, a greater degree of improvement in the spread of location errors could be observed between the results that correspond to the sets with N=10 and N=100, than N=100 and N=500.

### 3.2. Uncertainty Assessment of the Standard Gradient-Based Leak Localization Procedure

At the beginning, using dependency (15), an approximate estimation of the A type standard uncertainty of the individual measured pressures was made, depending on a number of N data samples. The mean values of these uncertainties obtained from all experiments are presented in Table 4. For each of the experiments, such an estimate was made for each of the three previously described time windows.

Analyzing the type A uncertainties presented in Table 4, one can observe that their values increase as the sample datasets are reduced in size.

It can also be noticed that the greatest uncertainty values correspond to the pressure measuring points closest to the pipeline inlet. For subsequent measurement points along the pipeline, the uncertainty values decreased. This can be justified by the fact that there are greater pressure oscillations in the initial section of the pipeline, which was also confirmed by the measurements for the state without leakage.

For datasets containing N=10 data samples, the values of the type A uncertainties, corresponding to the individual measured pressures, are not much lower than the uncertainties of type B, for which identical values equal to 0.49 kPa were adopted for all the measured pressures. For datasets containing N=100 data samples, the estimated type A uncertainty values are also significantly large. In both cases, this means that they cannot be omitted; however, in the case of sets containing N=500 data samples, the values of type A uncertainty are smaller by an order of magnitude, than those obtained for the measurement of the type B uncertainty. Hence, their impact has been neglected here.

For this reason, the resultant uncertainties corresponding to the individual measured pressures were calculated considering two components: uncertainties of type A as well as type B, according to Formula (14). In the case of the individual experiments, the values of the uncertainties of type A were estimated systematically on the basis of the data contained in a given time window.

Table 2 includes uncertainties, $u(z_{leak})$, estimated using dependency (11). Presented uncertainty values relate to each leak localization outcome and take into account all sensor pair configurations, “A”, “B”, and “C”, as well as the considered sets of data samples, N=10, N=100 and N=500.

It should be explained here that the uncertainties corresponding to the individual experiments show average values. They were calculated on the basis of the results obtained for three distinct time windows, similar to what was shown in the part related to the accuracy of leak localization.

When the calculated uncertainty of leak location, for any of the three time windows for each experiment, was greater than the assumed limit of 100 m; such cases were marked in bold. Moreover, when the calculated uncertainties were greater than 1000 m, their exact values were not given, but only the order determined by the number of the following character “*”.

In addition, Table 3 presents the averaged values of uncertainties for each leak location (marked as $u{\left(z_{leak}\right)}_{75}$, $u{\left(z_{leak}\right)}_{155}$ and $u{\left(z_{leak}\right)}_{235}$) and the averaged value for all of the locations together (marked as $u{\left(z_{leak}\right)}_{all}$).

By analyzing the results of the leak location uncertainty $u(z_{leak})$, we can observe:

- A significant increase of its value in the case of leaks with smaller and smaller sizes. This applies to leaks that were simulated in the pipeline’s initial and middle sections. In the case of the outlet section, the results are more varied;
- There were differences in the values obtained in the case of the use of different configurations of pressure sensors pairs. However, such differences are not so large as for the ones that are obtained for the leak location. This allows us to confirm the selection between the considered pressure sensor pair configurations, in the sense of their optimal use to locate leaks;
- There were differences in the values that relate to the use of the sets with the considered number N data samples. We can notice that such differences are greater between the considered N=10 and N=100 data sample sets, then between the ones with N=100 and N=500 data samples;
- For some experiments where the “B” sensor pair configuration was used, a very high leak location uncertainty was obtained. Such results are connected with the cases (marked in bold) when the calculated leak point goes beyond the extreme length coordinates of the pipeline;
- For the cases when the calculated leak point does not fall into the range defined by the coordinates corresponding to the internal pressure sensors used for the calculations of both pressure gradients (marked with bold characters), this is not visibly signaled in an increase in the uncertainty value.

To analyze all the above-discussed issues in detail, the so-called uncertainty budget calculated for standard leak localization procedure can be helpful.

Such a budget was elaborated using the example of an experiment with a leak simulated at the 155 m coordinate, of which the intensity was 1.20% of the nominal flow rate. From among the considered sensor pair configurations, the “C” configuration, as well as the pressure calculated on the basis of the N=100 sample datasets, which corresponded to one of the used time windows, were taken into account.

At the beginning, uncertainty budgets for determining the pressure gradients $G_{in}$ and $G_{out}$ were prepared. In case of the experiment in question, their budgets are presented in Table 5. As is mentioned above, the pressure values, $p_{in}$, $p_{n}$, $p_{m}$, $p_{out}$, which were used to calculate both pressure gradients, were the result of averaging N=100 data samples.

The analysis of uncertainty budgets for $G_{in}$ and $G_{out}$ pressure gradients determination shows that the uncertainty of pressure measurements has the greatest impact. The values of these shares are identical. The share of the distance between the pressure measurement points component is recognized as its value is smaller by an order of magnitude.

Table 6 presents the estimation of an uncertainty budget for the standard leak location procedure.

By analyzing the uncertainty budget presented in Table 6, it can be concluded that:

- The large leak location value of the uncertainty results from the large values of individual shares of uncertainty components. While comparing the individual contributors, the uncertainty share related to the length of the pipeline is negligible small. Uncertainty shares representing $G_{in}$ and $G_{out}$ gradients slightly differ from each other. This is due to the way their sensitivity coefficients are computed, which differ in using the value of the second gradient from the one for which the ratio is calculated. The uncertainty contributions for both gradients are approximately almost twice as large as the contributions for the pressure $p_{in}$ and $p_{out}$;
- The large values of the uncertainty shares related to gradients $G_{in}$ and $G_{out}$ and pressure $p_{in}$ and $p_{out}$ result from large values of their sensitivity coefficients. The calculation formulas of these coefficients are written as quotient, where the denominator is an expression with a value close to zero. This results from small differences between the $G_{in}$ and $G_{out}$ gradient values. These differences decrease along with the leak size decrease. In case of the sensitivity coefficients related to gradients, their differences are additionally squared, which brings the denominator value even closer to zero.

## 4. Conclusions

This article focuses on assessing the accuracy and uncertainty of a standard leak location procedure, based on pressure gradients, for liquid transmission pipelines. The scope of the assessment was focused on this procedure’s application to locate individual leaks. The evaluation was based on measurement data obtained from the model pipeline. First of all, we were interested in the accuracy and uncertainty of the location of small leaks with an intensity of 0.25–2.00% of the nominal flow rate. Leaks, which were simulated at the beginning, middle and end of the pipeline, were taken into account.

It is very important to assess the accuracy and uncertainty of the procedure in terms of the size of the analyzed leakage. Such levels of leakage often relate to thefts signaled by pipelines’ operators. However, for small leaks, the variations in the measured flow parameters are so small that they are often difficult to notice. This also applies to the discussed leak location procedure, which is based on the calculation of pressure gradients.

The verification of the procedure, carried out for the considered size and simulated leak positions, revealed a significant and considerable dispersion of the leak location accuracy results. It is worth mentioning that the procedure was implemented on the basis of averaged pressure values, calculated on the basis of sets containing different number of measurement samples. The research also considered three different configurations of sensor pairs, which were taken into account while calculating the pressure gradients. Particular configurations of such pairs differed in the distances between the sensors of a given pair and the degree of proximity (remoteness) to the location of leakage. It was found that individual configurations significantly affected the accuracy of the leak location. This was confirmed by the averaged location errors values, which were determined for each leakage spot. The best location accuracy was obtained in case of the configuration where the first pair consisted of a sensor located at the pipeline’s inlet and a sensor located closest to the leak, measured from the inlet of the pipeline, while the second pair consisted of a sensor located downstream closest to the leak and a sensor located at the outlet of the pipeline. In addition, a dependence was also observed in the form of a decrease in the accuracy of the leakage location for smaller and smaller leaks, but it was not a general rule.

The uncertainty estimation of the considered localization procedure was carried out according to the GUM convention. Systematic errors, as well as accidental errors, related to pressure measurements and distances between measurement points, were taken into account as the sources of uncertainty. The verification of the procedure’s uncertainty, carried out for the given size and simulated leaks’ locations, showed quite high values. It was observed that the appropriate selection of sensor pairs, included in the pressure gradients calculations, may significantly reduce the uncertainty level of the leak location results. Hence, the issue of the appropriate selection of sensor pairs becomes important. Additionally, for the considered sets containing different number of measurement samples used for the calculations of the nominal values of the individual pressures, differences in the results of the leak location uncertainty were also observed. In addition, one can also indicate the importance of further investigations in the field of uncertainty assessment.

## Figures and Tables

**Table 1:** Configurations of the pressure transmitters used for leak localization in conjunction with the pressure transmitters and the leak point positions. $G_{in}$ shows the position of the initial sensor pair (upstream from the leak point), while $G_{out}$ is the position of the final pair (downstream from the leak point), used in the gradient leak localization procedure in the carried out experiments and chosen depending on the coordinate of a simulated leak’s point.

Leak Position [m]	Configuration of Pairs of Transmitters Used for Calculation of Initial and Final Gradients		Pressure Transmitters—Position [m]					
			1	61	141	201	281	341
75	A	$G_{in}$	●	●				
		$G_{out}$					●	●
	B	$G_{in}$	●	●				
		$G_{out}$			●	●		
	C	$G_{in}$	●	●				
		$G_{out}$			●			●
155	A	$G_{in}$	●	●				
		$G_{out}$					●	●
	B	$G_{in}$		●	●			
		$G_{out}$				●	●	
	C	$G_{in}$	●		●			
		$G_{out}$				●		●
235	A	$G_{in}$	●	●				
		$G_{out}$					●	●
	B	$G_{in}$			●	●		
		$G_{out}$					●	●
	C	$G_{in}$	●			●		
		$G_{out}$					●	●

**Table 2:** Localization errors and uncertainties depending on the position and intensity of a leak, including the different number N of data samples used for pressure estimation, as well as following the sensor configuration A, B or C in the localization procedure. The following meaning is assigned to the marks: bold font—results go beyond the coordinates of the extreme sensors or are outside the pipeline; asterisks—the uncertainty achieves unreasonable high values.

Leaks		Localization Error [m]								
		Uncertainty [m]								
Position	Intensity	N = 10			N = 100			N = 500		
[m]	[%]	A	B	C	A	B	C	A	B	C
75	0.29	**−18.5**	−8.2	**−15.0**	**−14.2**	−11.3	**−18.5**	**−16.6**	−11.9	**−18.8**
		±36.9	±18.7	±15.2	±31.8	±16.3	±12.9	±31.4	±16.1	±12.7
	0.54	15.0	**−18.3**	**−17.5**	19.7	**−12.4**	**−13.6**	18.5	−12.0	−13.3
		±24.2	±16.7	±12.1	±22.5	±15.2	±11.3	±22.3	±14.9	±11.2
	0.83	44.6	**−11.3**	−4.2	48.1	−11.0	−5.3	47.0	−10.9	−6.7
		±20.0	±17.2	±11.3	±16.9	±14.4	±10.0	±17.1	±14.2	±10.2
	1.17	57.5	−10.1	−2.6	60.0	−8.5	−2.1	59.3	−8.4	−2.0
		±17.3	±16.1	±11.2	±14.6	±13.2	±9.2	±14.4	±13.0	±9.1
	1.29	59.3	−4.3	2.8	57.7	−7.6	0.1	58.5	−6.9	0.3
		±17.1	±15.8	±11.0	±14.6	±13.2	±9.1	±14.4	±13.0	±9.0
	1.93	57.2	−5.0	−0.7	53.3	−7.3	−2.7	51.6	−7.1	−2.8
		±15.3	±13.2	±9.5	±13.5	±11.5	±8.2	±13.3	±11.2	±8.1
155	0.24	−22.6	**132.1**	−2.9	−26.8	**209.7**	−3.7	−27.0	**199.9**	−4.2
		±19.9	******.	±22.6	±19.1	****.	±21.5	±19.2	±**845.3**	±21.5
	0.45	−47.2	**−7.2**	−10.5	−45.4	**−155.0**	−9.5	−45.6	**−155.0**	−10.4
		±24.8	*****.	±25.8	±21.3	****.	±22.4	±21.2	*****.	±22.3
	0.78	−54.8	**−119.9**	**−19.7**	−65.7	**−71.4**	**−15.3**	−64.0	**−89.5**	**−16.3**
		±25.3	±**403.3**	±24.6	±23.5	±**213.2**	±22.1	±22.9	±**237.2**	±21.8
	1.20	−17.0	**0.2**	3.3	−13.5	**−16.4**	−0.3	−11.2	**−16.6**	−0.3
		±19.4	±43.9	±16.1	±15.3	±45.5	±13.7	±15.3	±42.4	±13.4
	1.44	−3.1	**−33.1**	−1.4	−3.5	**−29.4**	−1.4	−4.4	**−27.4**	−0.6
		±16.1	±57.0	±14.8	±14.1	±46.5	±12.8	±13.6	±46.2	±12.4
	1.99	−15.6	**−18.9**	−2.2	−17.4	**−17.3**	−2.5	−16.2	**−21.9**	−4.3
		±15.7	±42.8	±13.6	±13.7	±35.0	±11.8	±13.5	±32.7	±11.5
235	0.37	−89.3	**44.6**	16.0	−91.4	41.6	13.8	−93.3	38.5	12.6
		±20.0	±34.9	±18.1	±18.4	±32.3	±16.9	±18.1	±31.4	±16.9
	0.54	**−180.9**	**128.6**	**−36.5**	**−174.9**	**−133.1**	−28.2	**−176.6**	**−192.4**	−26.3
		±34.1	****.	±71.7	±30.4	*****.	±57.4	±31.0	*****.	±60.3
	0.87	**−204.8**	**68.7**	**−90.3**	**−193.7**	**49.2**	**−58.3**	**−190.0**	**33.3**	**−49.2**
		±40.0	±**290.9**	±**150.3**	±34.8	±**295.4**	±**90.5**	±33.3	±**330.7**	±80.7
	1.28	−143.0	**48.4**	−7.4	−137.6	28.0	−5.9	−134.4	39.8	−1.1
		±27.5	±**142.3**	±37.2	±22.9	±70.2	±29.2	±22.5	±71.2	±27.8
	1.41	−128.7	**40.5**	1.8	−126.1	29.4	−0.2	−119.5	34.9	5.1
		±22.9	±66.1	±27.1	±21.3	±51.6	±24.7	±19.8	±44.4	±21.9
	1.85	−144.2	−10.9	−21.1	−137.0	9.0	−13.0	−138.0	7.8	−13.6
		±23.8	±57.7	±32.4	±21.7	±58.2	±27.4	±21.6	±58.3	±27.6

**Table 3:** Averaged absolute values of localization errors and averaged values of localization uncertainties depending on the leak location, including the different number N of data samples used for pressure estimation, as well as following the sensor configuration A, B or C in the localization procedure.

Leaks: In Relation to Leak Location	Absolute Values of Localization Error [m]								
	Localization Uncertainty [m]								
	N = 10			N = 100			N = 500		
	A	B	C	A	B	C	A	B	C
${\left|error\right|}_{75}$	42.0	9.5	7.4	42.2	9.7	7.1	41.9	9.5	7.3
$u{\left(z_{leak}\right)}_{75}$	±21.8	±16.3	±11.7	±19.0	±14.0	±10.1	±18.8	±13.7	±10.0
${\left|error\right|}_{155}$	27.1	79.5	7.4	28.7	83.2	5.6	28.0	85.1	6.0
$u{\left(z_{leak}\right)}_{155}$	±20.2	–	±19.6	±17.8	–	±17.4	±17.6	–	±17.1
${\left|error\right|}_{235}$	148.5	56.9	29.2	143.5	56.2	20.1	142.0	57.8	18.1
$u{\left(z_{leak}\right)}_{235}$	±28.1	–	±56.1	±24.9	–	±41.0	±24.4	–	±39.2
${\left|error\right|}_{all}$	72.5	48.6	14.7	71.5	49.7	10.9	70.6	50.8	10.5
$u{\left(z_{leak}\right)}_{all}$	±23.4	–	±29.1	±20.6	–	±22.8	±20.3	–	±22.1

**Table 4:** Approximated results of standard uncertainties of type A.

Number of Samples	Standard Deviation of Mean of Measured Pressures [kPa] (at the Points in the Pipeline with the Coordinates [m])					
	1	61	141	201	281	341
N=10	0.34	0.31	0.27	0.25	0.25	0.25
N=100	0.12	0.10	0.09	0.09	0.09	0.08
N=500	0.05	0.05	0.04	0.04	0.04	0.04

**Table 5:** Uncertainty budget for $G_{in}$ and $G_{out}$ gradients determination.

Process Variable	Value	Uncertainty $u(x_{i})$	Sensitivity Coefficient		Uncertainty Component $u(x_{i})c(x_{i})$
			Formula	Value	
gradient $G_{in}$					
$p_{n}$	491.58 kPa	0.50 kPa	$1/z_{in,n}$	0.0071 m^−1^	0.0036 kPa/m
$p_{in}$	755.98 kPa	0.50 kPa	$-1/z_{in,n}$	−0.0071 m^−1^	−0.0036 kPa/m
$z_{in,n}$	140 m	0.025 m	$-(p_{n}-p_{in})/z_{in,n}^{2}$	0.0135 kPa/m^2^	0.0003 kPa/m
$G_{in}$	−1.8886 kPa/m	$u\left(G_{in}\right)$ = 0.0050 kPa/m			
gradient $G_{out}$					
$p_{out}$	133.12 kPa	0.50 kPa	$-1/z_{m,out}$	−0.0071 m^−1^	−0.0036 kPa/m
$p_{m}$	383.10 kPa	0.50 kPa	$1/z_{m,out}$	0.0071 m^−1^	0.0036 kPa/m
$z_{m,out}$	140 m	0.025 m	$-(p_{out}-p_{m})/z_{m,out}^{2}$	0.0128 kPa/m^2^	0.0003 kPa/m
$G_{out}$	−1.7856 kPa/m	$u\left(G_{out}\right)$ = 0.0050 kPa/m			

**Table 6:** Uncertainty budget for leak location determination.

Process Variable	Value	Uncertainty $u(x_{i})$	Sensitivity Coefficient		Uncertainty Component $u(x_{i})c(x_{i})$
			Formula	Value	
L	340 m	0.025 m	$\frac{-G_{out}}{G_{in}-G_{out}}$	−17.3	−0.4 m
$G_{in}$	−1.8886 kPa/m	0.0050 kPa/m	$\frac{-(p_{out}-p_{in}-G_{out}\cdot L)}{{\left(G_{in}-G_{out}\right)}^{2}}$	1485.2 m^2^/kPa	7.4 m
$G_{out}$	−1.7856 kPa/m	0.0050 kPa/m	$\frac{p_{out}-p_{in}-G_{in}\cdot L}{{\left(G_{in}-G_{out}\right)}^{2}}$	1815.8 m^2^/kPa	9.1 m
$p_{in}$	755.98 kPa	0.50 kPa	$\frac{-1}{G_{in}-G_{out}}$	9.7 m/kPa	4.9 m
$p_{out}$	133.12 kPa	0.50 kPa	$\frac{1}{G_{in}-G_{out}}$	−9.7 m/kPa	−4.9 m
$z_{leak}$	154.0 m	$u\left(z_{leak}\right)$ = 13.6 m			

## Data Availability

Not applicable.
